# Headaches from Eye Strain

**Published:** 1897-12

**Authors:** 


### Headaches from Eye Strain.

Dr. S. Weir Mitchell formulates the following conclusions: There are many headaches which are due directly to disorders of the refractive or accommodative apparatus of the eyes. In some instances the brain symptom is often the most prominent and sometimes the sole prominent symptom of the eye troubles, so that while there may be no pain or sense of fatigue in the eye, the strain with which it is used may be interpreted solely by occipital or frontal headache. The long continuance of eye troubles may be the unsuspected source of insomnia, vertigo, nausea, and general failure of health. In many cases the eye trouble becomes suddenly mischievous, owing to some failure of the general health, or to increased sensitiveness of the brain from moral or mental causes.—*Practical Druggist.*

To remove blood stains soak in a twenty-five percent solution of potassium iodide.

